# Supplementary material for: Alterations in Circulating Bile Acids in Metabolic Dysfunction-Associated Steatotic Liver Disease: A Systematic Review and Meta-Analysis
Source: Biomolecules. 2023 Sep 6;13(9):1356. doi: 10.3390/biom13091356 (PMC10526305; doi:10.3390/biom13091356)
Supplement: Supplementary file 1 [file biomolecules-13-01356-s001.zip › biomolecules-2576257-supplementary.pdf]

biomolecules

MDPI

Systematic Review

Alterations in Circulating Bile Acids in Metabolic Dysfunction-Associated Steatotic Liver Disease: A Systematic Review and Meta-Analysis

Jiaming Lai †, Ling Luo †, Ting Zhou, Xiongcai Feng, Junzhao Ye * and Bihui Zhong *

Department of Gastroenterology of the First Affiliated Hospital, Sun Yat-sen University, No. 58 Zhongshan II Road, Yuexiu District, Guangzhou 510080, China; laijm6@mail2.sysu.edu.cn (J.L.); luol27@mail2.sysu.edu.cn (L.L.); zhouting_blawan@163.com (T.Z.); fengxc3@mail2.sysu.edu.cn (X.F.)

* Correspondence: yejzh@mail2.sysu.edu.cn (J.Y.); zhongbh@mail.sysu.edu.cn (B.Z.); Tel./Fax: +(8620)-87766335 (J.Y. and B.Z.)

† These authors contributed equally to this work.

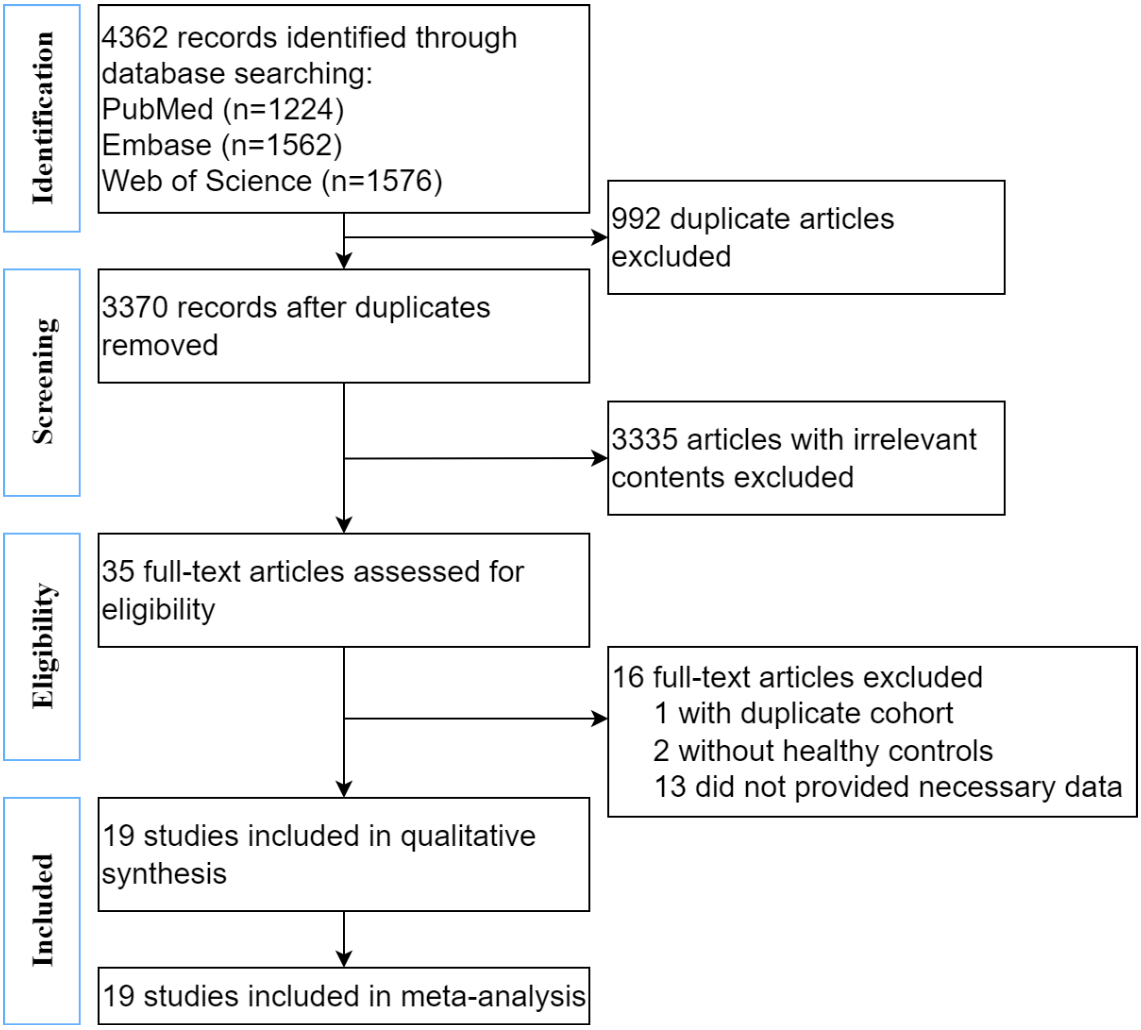

Figure S1. Meta-analysis diagram of study selection flow.

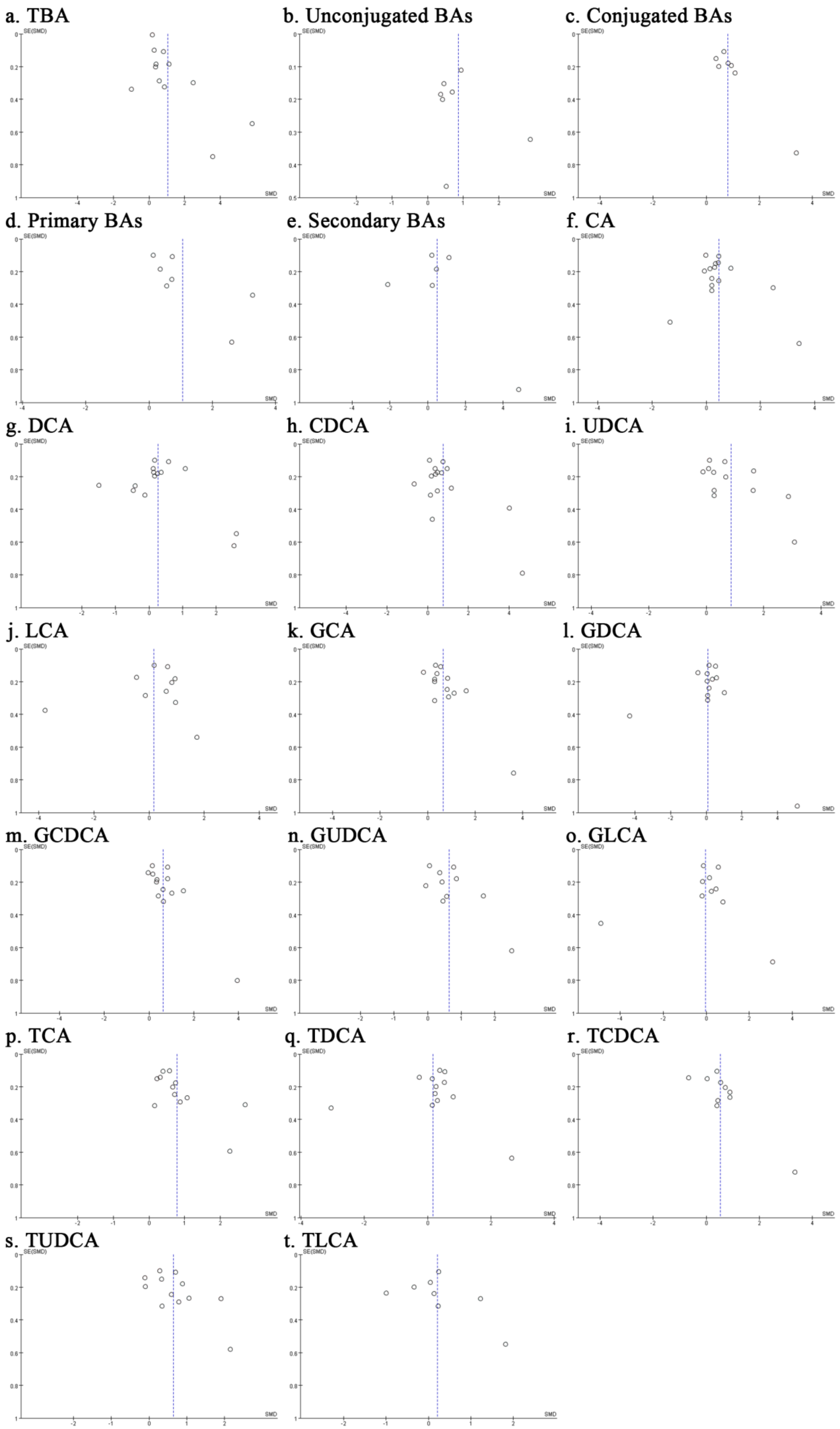
a. TBA
b. Unconjugated BAs
c. Conjugated BAs
d. Primary BAs
e. Secondary BAs
f. CA
g. DCA
h. CDCA
i. UDCA
j. LCA
k. GCA
l. GDCA
m. GCDCA
n. GUDCA
o. GLCA
p. TCA
q. TDCA
r. TCDCA
s. TUDCA
t. TLCA
SE(SMD)
SMD

Figure S2. Funnel plots estimate the potential publication bias of the included studies. a. TBA, total bile acid; b. unconjugated BAs; c. conjugated BAs; d. primary BAs; e. secondary BAs; f. CA, cholic acid; g. DCA, deoxycholic acid; h. CDCA, chenodeoxycholic acid; i. UDCA, ursodeoxycholic acid; j. LCA, lithocholic acid; k. GCA, glycocholic acid; l. GDCA, glycodeoxycholic acid; m. GCDCA, glycochenodeoxycholic acid; n. GUDCA, glycoursodeoxycholic acid; o. GLCA, glycolithocholic acid; p. TCA, taurococholic acid; q. TDCA, taurodeoxycholic acid; r. TCDCA, taurochenodeoxycholic acid; s. TUDCA, tauroursodeoxycholic acid; t. TLCA, taurolithocholic acid.

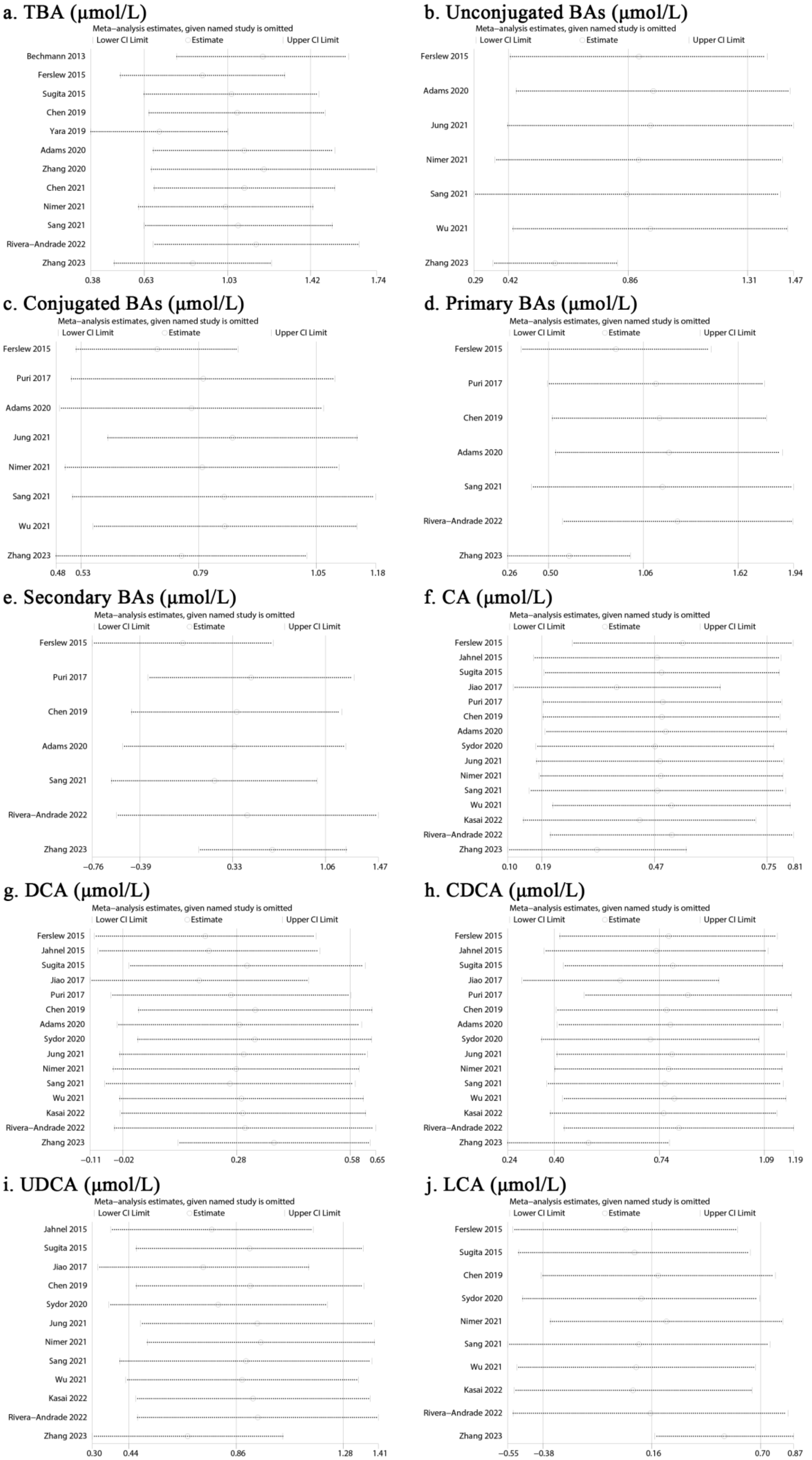

a. TBA (μmol/L)
Meta–analysis estimates, given named study is omitted
Lower CI Limit
Estimate
Upper CI Limit
Bechmann 2013
Ferslew 2015
Sugita 2015
Chen 2019
Yara 2019
Adams 2020
Zhang 2020
Chen 2021
Nimer 2021
Sang 2021
Rivera–Andrade 2022
Zhang 2023
0.38 0.63 1.03 1.42 1.74
b. Unconjugated BAs (μmol/L)
Meta–analysis estimates, given named study is omitted
Lower CI Limit
Estimate
Upper CI Limit
Ferslew 2015
Adams 2020
Jung 2021
Nimer 2021
Sang 2021
Wu 2021
Zhang 2023
0.29 0.42 0.86 1.31 1.47
c. Conjugated BAs (μmol/L)
Meta–analysis estimates, given named study is omitted
Lower CI Limit
Estimate
Upper CI Limit
Ferslew 2015
Puri 2017
Adams 2020
Jung 2021
Nimer 2021
Sang 2021
Wu 2021
Zhang 2023
0.48 0.53 0.79 1.05 1.18
d. Primary BAs (μmol/L)
Meta–analysis estimates, given named study is omitted
Lower CI Limit
Estimate
Upper CI Limit
Ferslew 2015
Puri 2017
Chen 2019
Adams 2020
Sang 2021
Rivera–Andrade 2022
Zhang 2023
0.26 0.50 1.06 1.62 1.94
e. Secondary BAs (μmol/L)
Meta–analysis estimates, given named study is omitted
Lower CI Limit
Estimate
Upper CI Limit
Ferslew 2015
Puri 2017
Chen 2019
Adams 2020
Sang 2021
Rivera–Andrade 2022
Zhang 2023
−0.76 −0.39 0.33 1.06 1.47
f. CA (μmol/L)
Meta–analysis estimates, given named study is omitted
Lower CI Limit
Estimate
Upper CI Limit
Ferslew 2015
Jahnel 2015
Sugita 2015
Jiao 2017
Puri 2017
Chen 2019
Adams 2020
Sydor 2020
Jung 2021
Nimer 2021
Sang 2021
Wu 2021
Kasai 2022
Rivera–Andrade 2022
Zhang 2023
0.10 0.19 0.47 0.75 0.81
g. DCA (μmol/L)
Meta–analysis estimates, given named study is omitted
Lower CI Limit
Estimate
Upper CI Limit
Ferslew 2015
Jahnel 2015
Sugita 2015
Jiao 2017
Puri 2017
Chen 2019
Adams 2020
Sydor 2020
Jung 2021
Nimer 2021
Sang 2021
Wu 2021
Kasai 2022
Rivera–Andrade 2022
Zhang 2023
−0.11 −0.02 0.28 0.58 0.65
h. CDCA (μmol/L)
Meta–analysis estimates, given named study is omitted
Lower CI Limit
Estimate
Upper CI Limit
Ferslew 2015
Jahnel 2015
Sugita 2015
Jiao 2017
Puri 2017
Chen 2019
Adams 2020
Sydor 2020
Jung 2021
Nimer 2021
Sang 2021
Wu 2021
Kasai 2022
Rivera–Andrade 2022
Zhang 2023
0.24 0.40 0.74 1.09 1.19
i. UDCA (μmol/L)
Meta–analysis estimates, given named study is omitted
Lower CI Limit
Estimate
Upper CI Limit
Jahnel 2015
Sugita 2015
Jiao 2017
Chen 2019
Sydor 2020
Jung 2021
Nimer 2021
Sang 2021
Wu 2021
Kasai 2022
Rivera–Andrade 2022
Zhang 2023
0.30 0.44 0.86 1.28 1.41
j. LCA (μmol/L)
Meta–analysis estimates, given named study is omitted
Lower CI Limit
Estimate
Upper CI Limit
Ferslew 2015
Sugita 2015
Chen 2019
Sydor 2020
Nimer 2021
Sang 2021
Wu 2021
Kasai 2022
Rivera–Andrade 2022
Zhang 2023
−0.55 −0.38 0.16 0.70 0.87

Figure S3. Sensitivity analysis chart was included in the study. a. TBA, total bile acid; b. unconjugated BAs; c. conjugated BAs; d. primary BAs; e. secondary BAs; f. CA, cholic acid; g. DCA, deoxycholic acid; h. CDCA, chenodeoxycholic acid; i. UDCA, ursodeoxycholic acid; j. LCA, lithocholic acid.

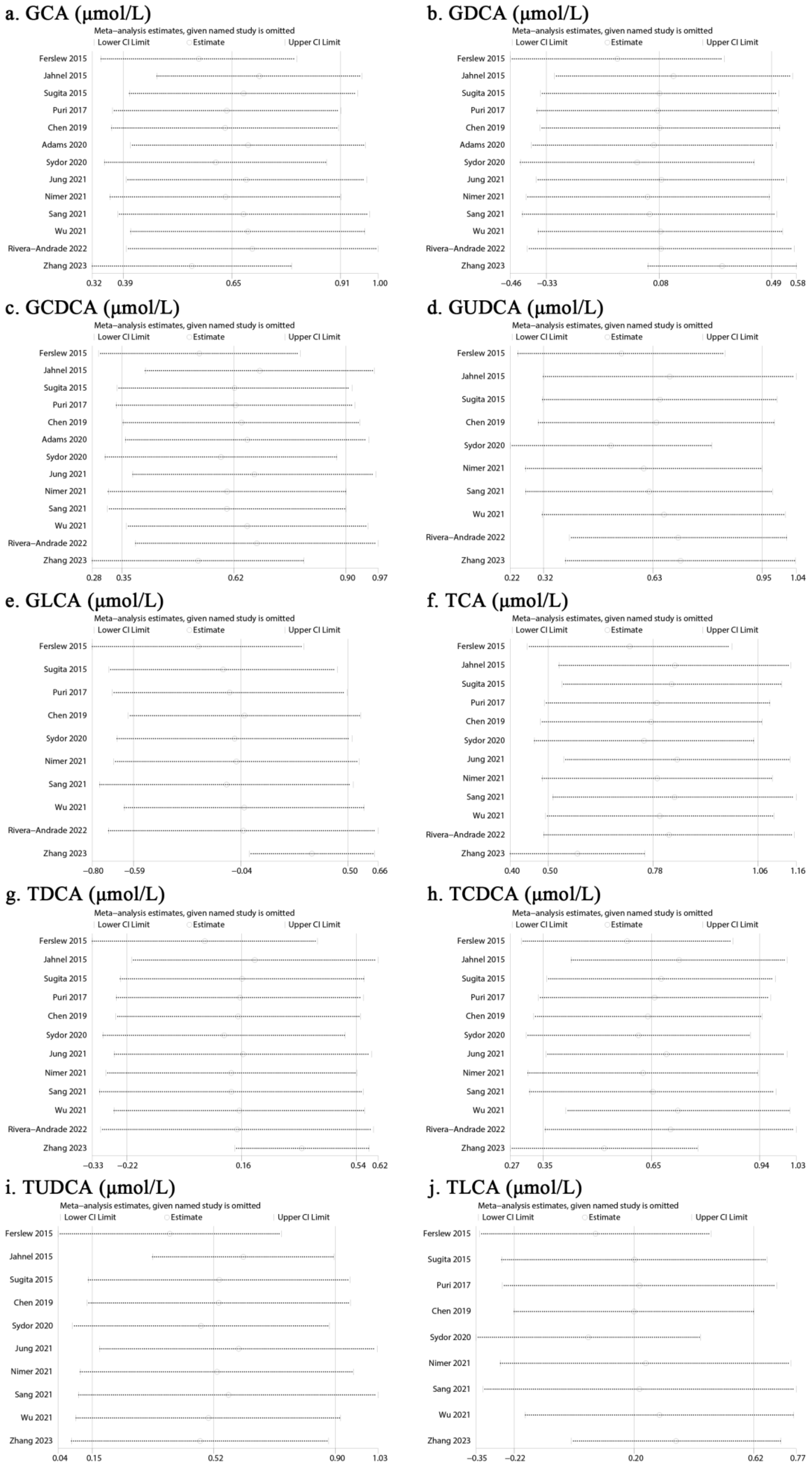

a. GCA (μmol/L)
Meta−analysis estimates, given named study is omitted
Lower CI Limit
Estimate
Upper CI Limit
Ferslew 2015
Jahnel 2015
Sugita 2015
Puri 2017
Chen 2019
Adams 2020
Sydor 2020
Jung 2021
Nimer 2021
Sang 2021
Wu 2021
Rivera−Andrade 2022
Zhang 2023
0.32 0.39 0.65 0.91 1.00
b. GDCA (μmol/L)
Meta−analysis estimates, given named study is omitted
Lower CI Limit
Estimate
Upper CI Limit
Ferslew 2015
Jahnel 2015
Sugita 2015
Puri 2017
Chen 2019
Adams 2020
Sydor 2020
Jung 2021
Nimer 2021
Sang 2021
Wu 2021
Rivera−Andrade 2022
Zhang 2023
−0.46 −0.33 0.08 0.49 0.58
c. GCDCA (μmol/L)
Meta−analysis estimates, given named study is omitted
Lower CI Limit
Estimate
Upper CI Limit
Ferslew 2015
Jahnel 2015
Sugita 2015
Puri 2017
Chen 2019
Adams 2020
Sydor 2020
Jung 2021
Nimer 2021
Sang 2021
Wu 2021
Rivera−Andrade 2022
Zhang 2023
0.28 0.35 0.62 0.90 0.97
d. GUDCA (μmol/L)
Meta−analysis estimates, given named study is omitted
Lower CI Limit
Estimate
Upper CI Limit
Ferslew 2015
Jahnel 2015
Sugita 2015
Chen 2019
Sydor 2020
Nimer 2021
Sang 2021
Wu 2021
Rivera−Andrade 2022
Zhang 2023
0.22 0.32 0.63 0.95 1.04
e. GLCA (μmol/L)
Meta−analysis estimates, given named study is omitted
Lower CI Limit
Estimate
Upper CI Limit
Ferslew 2015
Sugita 2015
Puri 2017
Chen 2019
Sydor 2020
Nimer 2021
Sang 2021
Wu 2021
Rivera−Andrade 2022
Zhang 2023
−0.80 −0.59 −0.04 0.50 0.66
f. TCA (μmol/L)
Meta−analysis estimates, given named study is omitted
Lower CI Limit
Estimate
Upper CI Limit
Ferslew 2015
Jahnel 2015
Sugita 2015
Puri 2017
Chen 2019
Sydor 2020
Jung 2021
Nimer 2021
Sang 2021
Wu 2021
Rivera−Andrade 2022
Zhang 2023
0.40 0.50 0.78 1.06 1.16
g. TDCA (μmol/L)
Meta−analysis estimates, given named study is omitted
Lower CI Limit
Estimate
Upper CI Limit
Ferslew 2015
Jahnel 2015
Sugita 2015
Puri 2017
Chen 2019
Sydor 2020
Jung 2021
Nimer 2021
Sang 2021
Wu 2021
Rivera−Andrade 2022
Zhang 2023
−0.33 −0.22 0.16 0.54 0.62
h. TCDCA (μmol/L)
Meta−analysis estimates, given named study is omitted
Lower CI Limit
Estimate
Upper CI Limit
Ferslew 2015
Jahnel 2015
Sugita 2015
Puri 2017
Chen 2019
Sydor 2020
Jung 2021
Nimer 2021
Sang 2021
Wu 2021
Rivera−Andrade 2022
Zhang 2023
0.27 0.35 0.65 0.94 1.03
i. TUDCA (μmol/L)
Meta−analysis estimates, given named study is omitted
Lower CI Limit
Estimate
Upper CI Limit
Ferslew 2015
Jahnel 2015
Sugita 2015
Chen 2019
Sydor 2020
Jung 2021
Nimer 2021
Sang 2021
Wu 2021
Zhang 2023
0.04 0.15 0.52 0.90 1.03
j. TLCA (μmol/L)
Meta−analysis estimates, given named study is omitted
Lower CI Limit
Estimate
Upper CI Limit
Ferslew 2015
Sugita 2015
Puri 2017
Chen 2019
Sydor 2020
Nimer 2021
Sang 2021
Wu 2021
Zhang 2023
−0.35 −0.22 0.20 0.62 0.77

Figure S4. Sensitivity analysis chart was included in the study. a. GCA, glycocholic acid; b. GDCA, glycodeoxycholic acid; c. GCDCA, glycochenodeoxycholic acid; d. GUDCA, glycoursodeoxycholic acid; e. GLCA, glycolithocholic acid; f. TCA, taurocholic acid; g. TDCA, taurodeoxycholic acid; h. TCDCA, taurochenodeoxycholic acid; i. TUDCA, tauroursodeoxycholic acid; j. TLCA, taurolithocholic acid.

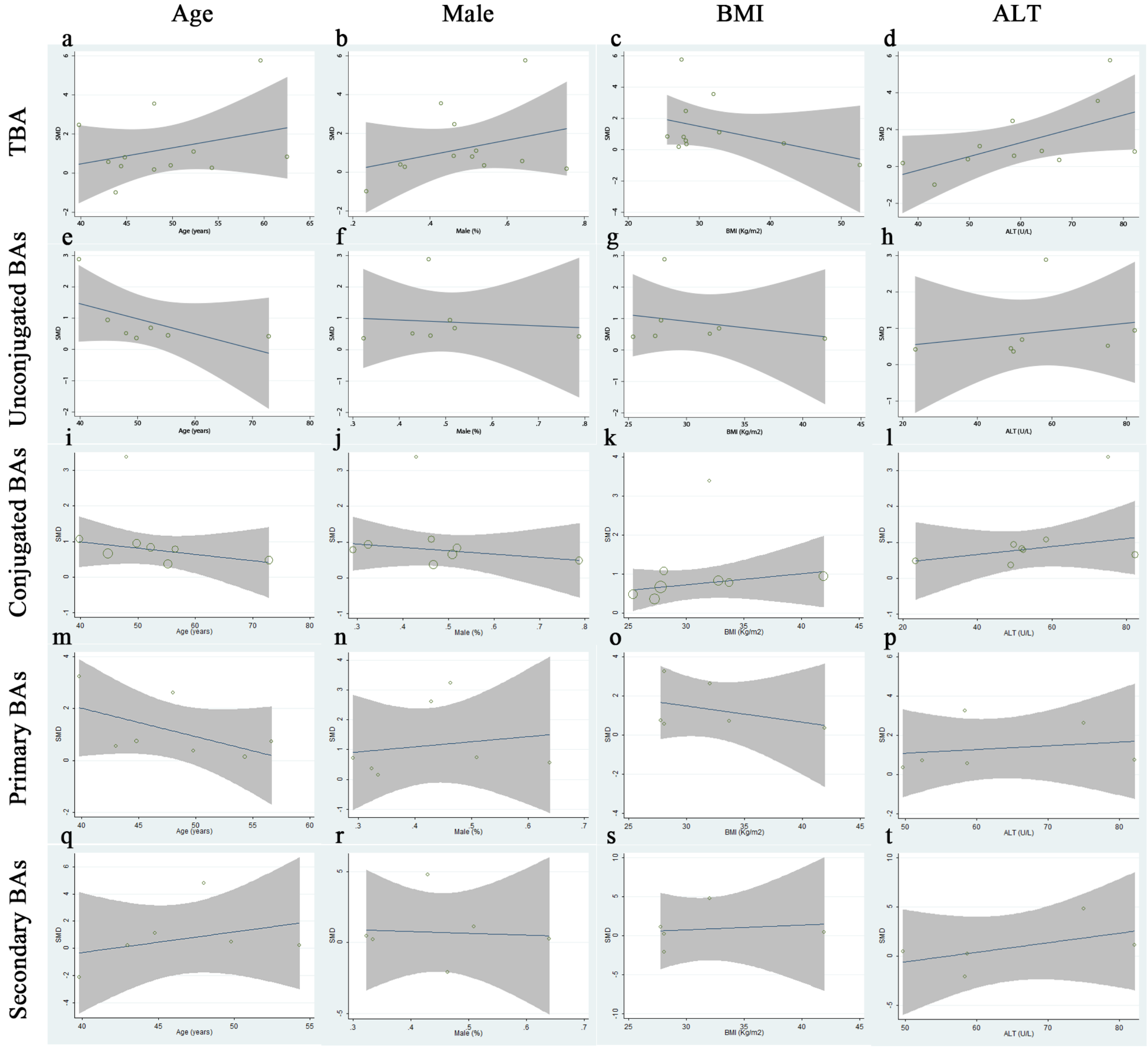

Figure S5. Meta-regression analyses for the effect of age, the male ratio, BMI, or ALT on TBA (a-d), unconjugated (e-h) and conjugated (i-l) BAs, and primary (m-p) and secondary (q-t) BAs concentration changes in MASLD patients. Each data point overlaps to form a circle. The size of a circle represents the weight of the corresponding data point, and the larger the circle, the greater the impact. Abbreviations: BMI, body mass index; ALT, alanine aminotransferase; TBA, total bile acid; BA, bile acid.

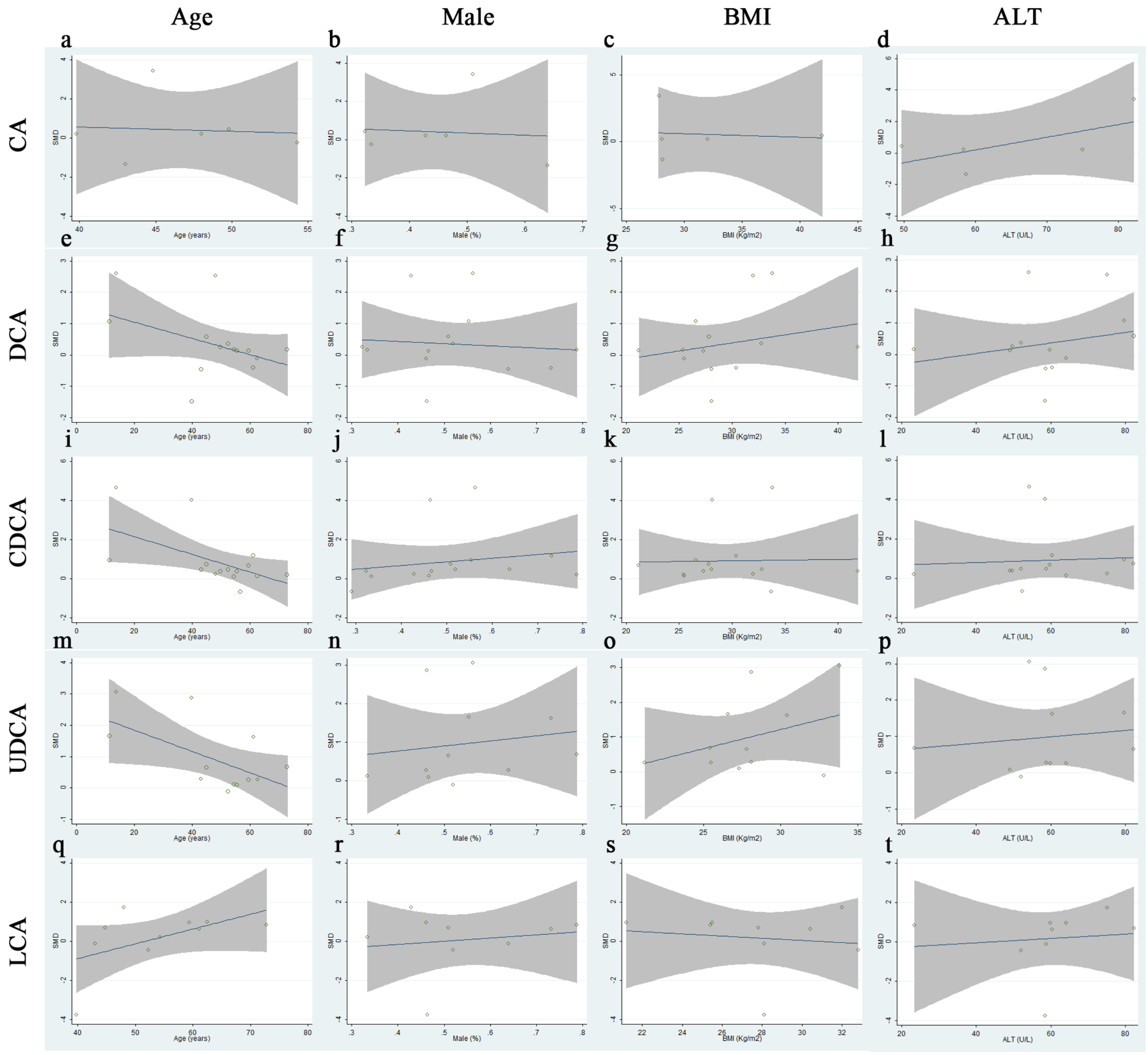

Figure S6. Meta-regression analyses for the effect of age, the male ratio, BMI, or ALT on CA (a-d), DCA (e-h), CDCA (i-l), UDCA (m-p), and LCA (q-t) concentration changes in MASLD patients. Each data point overlaps to form a circle. The size of a circle represents the weight of the corresponding data point, and the larger the circle, the greater the impact. Abbreviations: BMI, body mass index; ALT, alanine aminotransferase; CA, cholic acid; DCA, deoxycholic acid; CDCA, chenodeoxycholic acid; UDCA, ursodeoxycholic acid; LCA, lithocholic acid.

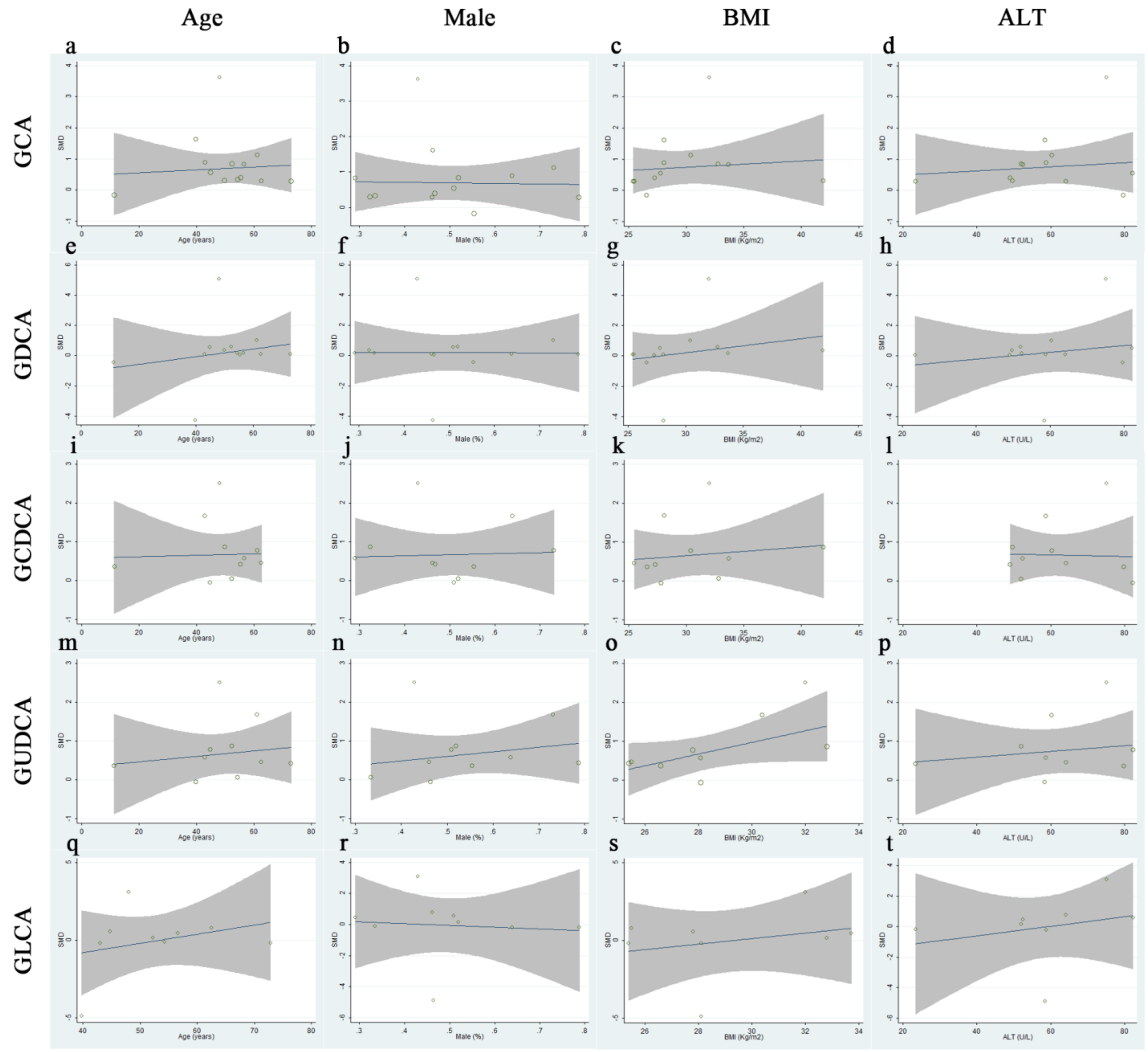

Figure S7. Meta-regression analyses for the effect of age, the male ratio, BMI, or ALT on GCA (a-d), GDCA (e-h), GCDCA (i-l), GUDCA (m-p), and GLCA (q-t) concentration changes in MASLD patients. Each data point overlaps to form a circle. The size of a circle represents the weight of the corresponding data point, and the larger the circle, the greater the impact. Abbreviations: BMI, body mass index; ALT, alanine aminotransferase; GCA, glycocholic acid; GDCA, glycodeoxycholic acid; GCDCA, glycochenodeoxycholic acid; GUDCA, glycoursodeoxycholic acid; GLCA, glycolithocholic acid.

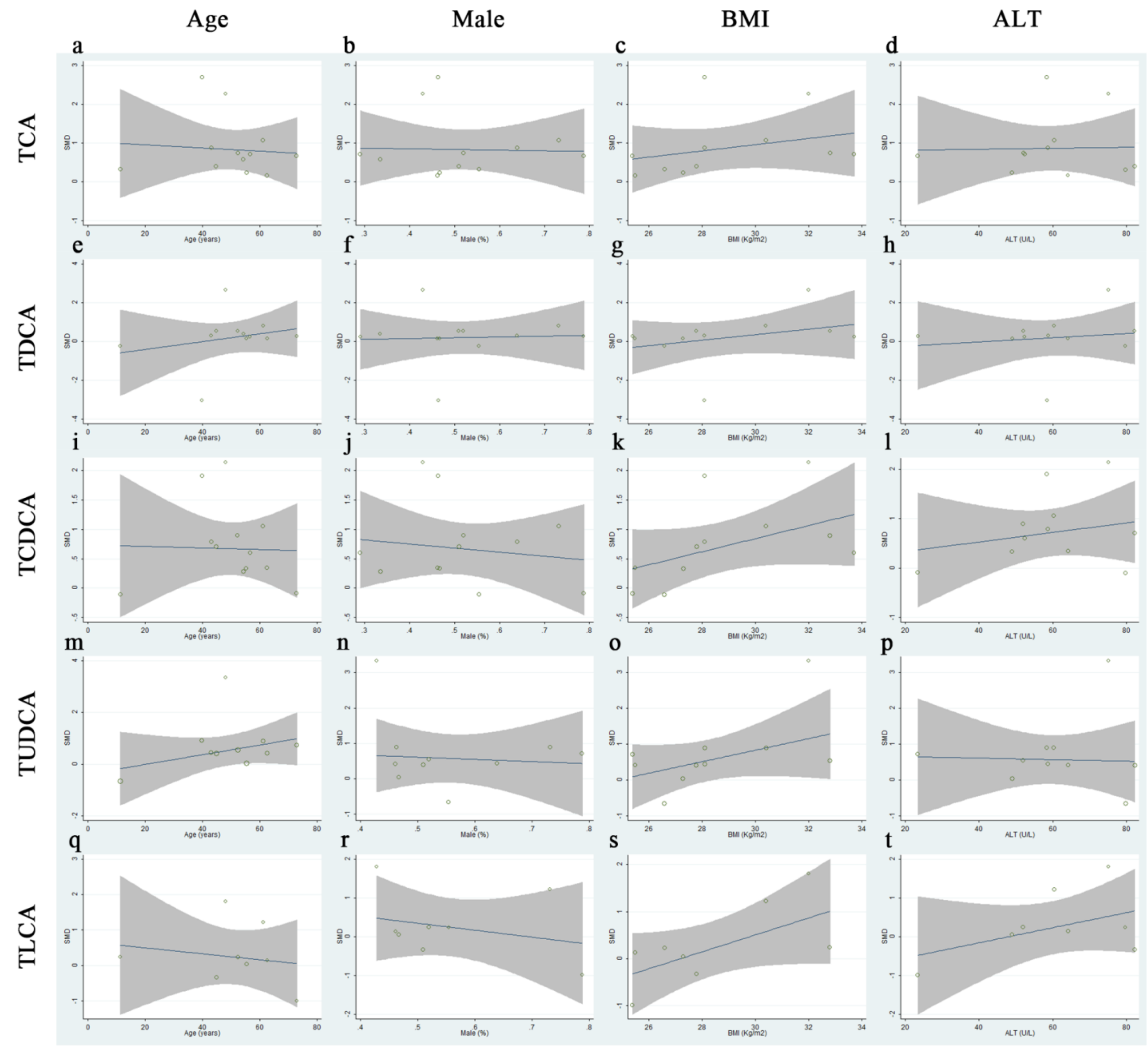

Figure S8. Meta-regression analyses for the effect of age, the male ratio, BMI, or ALT on TCA (a-d), TDCA (e-h), TCDCA (i-l), TUDCA (m-p), and TLCA (q-t) concentration changes in MASLD patients. Each data point overlaps to form a circle. The size of a circle represents the weight of the corresponding data point, and the larger the circle, the greater the impact. Abbreviations: BMI, body mass index; ALT, alanine aminotransferase; TCA, taurocholic acid; TDCA, taurodeoxycholic acid; TCDCA, taurochenodeoxycholic acid; TUDCA, tauroursodeoxycholic acid; TLCA, taurolithocholic acid.

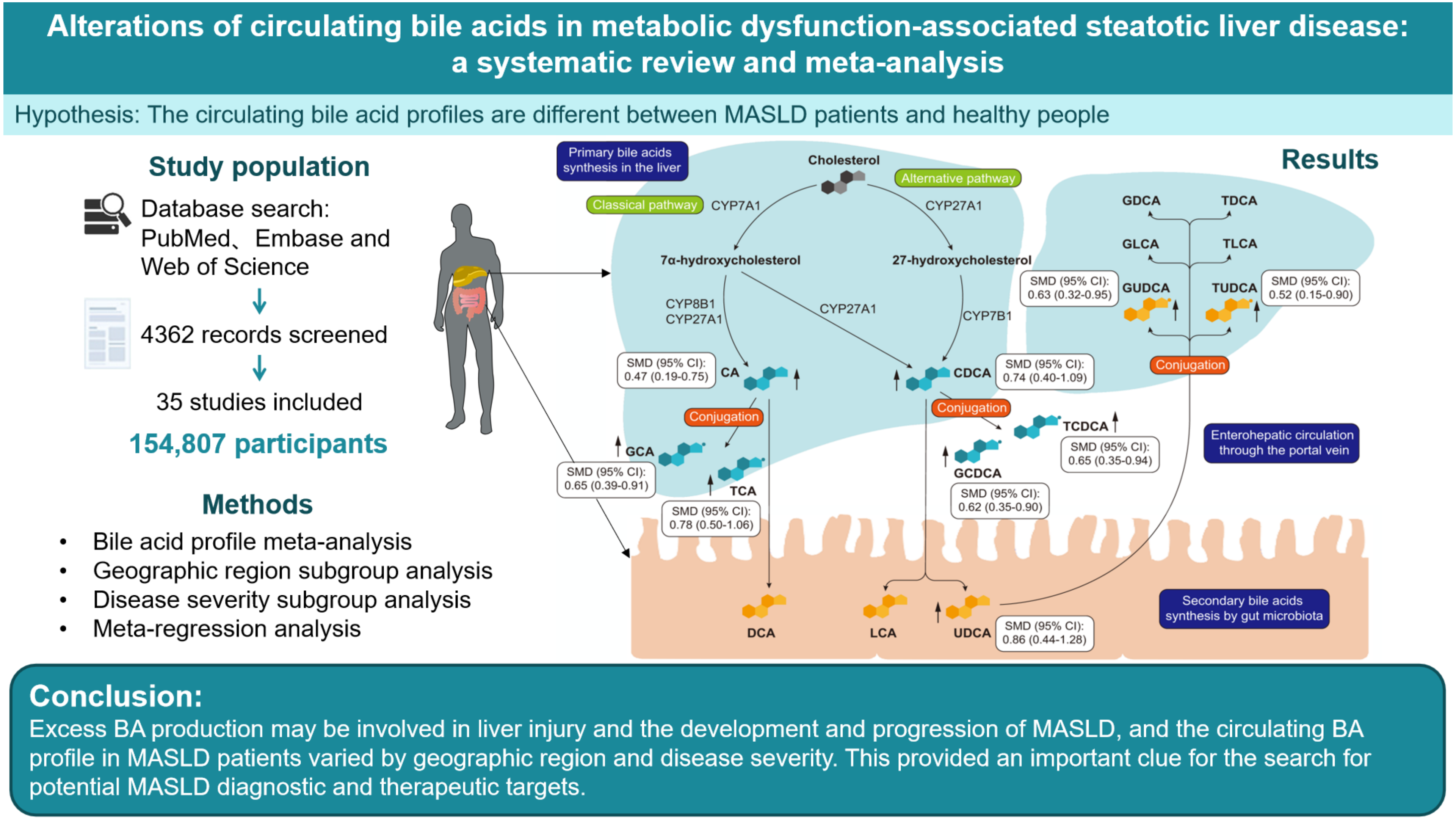

Figure S9. Graphical summary

Table S1. Detailed Search Strategy.

Source	Search strategy
PubMed	1: "Bile Acids and Salts"[Mesh]
	2: "Bile Acids"
	3: "Acids, Bile"
	4: "Bile Salts"
	5: "Salt, Bile"
	6: 1 OR 2 OR 3 OR 4 OR 5
	7: "Fatty Liver"[Mesh]
	8: Steatohepatitis
	9: "Steatosis of Liver"
	10: "Liver Steatosis"
	11: 7 OR 8 OR 9 OR 10
	12: "Humans"[Mesh]
	13: 6 AND 11 AND 12
Embase	1: 'fatty liver'/exp/mj
	2: steatohepatitis
	3: 'steatosis of liver'
	4: 'liver steatosis'
	5: 'fatty liver'
	6: 1 OR 2 OR 3 OR 4 OR 5
	7: 'bile acid'/exp/mj
	8: 'bile acid'
	9: 'acids, bile'
	10: 'bile salts'
	11:'salt, bile'
	12: 7 OR 8 OR 9 OR 10 OR 11
	13: 'human'/exp
	14: 6 AND 12 AND 13
Web of Science	1: "bile acid"
	2: "Bile Salts"
	3: "Salt, Bile"
	4: 1 OR 2 OR 3

	5: "fatty liver"
	6: "Steatohepatitis"
	7: "Steatosis of Liver"
	8: "Liver Steatosis"
	9: 5 OR 6 OR 7 OR 8
	10: mice
	11: mouse
	12: cow
	13: goose
	14: hamster
	15: NOT 10 OR 11 OR 12 OR 13 OR 14
	16: 4 AND 9 AND 15

Table S2. Quality assessment of included studies using the Newcastle–Ottawa scale (NOS) (case-control studies).

Study	Selection	Comparability	Exposure	Score
Bechmann 2013 [17]	4	1	2	7
Ferslew 2015 [18]	4	2	2	8
Jahnel 2015 [19]	4	2	2	8
Sugita 2015 [20]	4	0	2	6
Jiao 2017 [21]	4	2	2	8
Puri 2017 [22]	4	1	2	7
Chen 2019 [23]	4	2	2	8
Yara 2019 [24]	4	2	2	8
Adams 2020 [25]	4	2	2	8
Sydor 2020 [26]	4	1	2	7
Zhang 2020 [27]	4	2	2	8
Chen 2021 [28]	4	2	2	8
Jung 2021 [29]	4	1	2	7
Nimer 2021 [30]	4	0	2	6
Sang 2021 [31]	4	2	2	8
Wu 2021 [32]	4	1	2	7
Kasai 2022 [33]	4	1	2	7
Rivera-Andrade 2022 [34]	4	1	2	7
Zhang 2023 [35]	4	0	2	6

Table S3. ***p*****-value for Begg's and Egger's tests for publication bias.**

**Characteristics**	**Study (n)**	***P* for Begg's test**	***P* for Egger's test**
Total bile acids	12	0.244	0.017
Total unconjugated bile acids	7	1.000	0.619
Total conjugated bile acids	8	0.108	0.039
Total primary bile acids	7	0.133	0.102
Total secondary bile acids	6	1.000	0.863
Cholic acid (CA)	15	1.000	0.325
Deoxycholic acid (DCA)	14	0.913	0.897
Chenodeoxycholic acid (CDCA)	15	0.488	0.141
Ursodeoxycholic acid (UDCA)	12	0.064	0.088
Lithocholic acid (LCA)	10	0.721	0.584
Glycocholic acid (GCA)	13	0.059	0.029
Glycodeoxycholic acid (GDCA)	13	0.951	0.743
Glycochenodeoxycholic acid (GCDCA)	13	0.077	0.054
Glycoursodeoxycholic acid (GUDCA)	10	0.283	0.164
Glycolithocholic acid (GLCA)	9	0.754	0.622
Taurococholic acid (TCA)	12	0.034	0.039
Taurodeoxycholic acid (TDCA)	12	0.945	0.595
Taurochenodeoxycholic acid (TCDCA)	12	0.150	0.133
Tauroursodeoxycholic acid (TUDCA)	10	0.152	0.107
Taurolithocholic acid (TLCA)	8	0.386	0.683

Table S4: Results of the GRADE assessment for each outcome.

	Risk of Bias	Inconsistency	Indirectness	Imprecision	Publication Bias	Certainty of evidence
Total bile acids	No	Yes (2x)	No	No	No	Very low
Total unconjugated bile acids	No	Yes (2x)	No	No	No	Very low
Total conjugated bile acids	No	Yes (2x)	No	No	No	Very low
Total primary bile acids	No	Yes (2x)	No	No	No	Very low
Total secondary bile acids	No	Yes (2x)	No	No	No	Very low
Cholic acid (CA)	No	Yes (2x)	No	No	No	Very low
Deoxycholic acid (DCA)	No	Yes (2x)	No	No	No	Very low
Chenodeoxycholic acid (CDCA)	No	Yes (2x)	No	No	No	Very low
Ursodeoxycholic acid (UDCA)	No	Yes (2x)	No	No	No	Very low
Lithocholic acid (LCA)	No	Yes (2x)	No	No	No	Very low
Glycocholic acid (GCA)	No	Yes (2x)	No	No	No	Very low
Glycodeoxycholic acid (GDCA)	No	Yes (2x)	No	No	No	Very low
Glycochenodeoxycholic acid (GCDCA)	No	Yes (2x)	No	No	No	Very low
Glycoursodeoxycholic acid (GUDCA)	No	Yes (2x)	No	No	No	Very low
Glycolithocholic acid (GLCA)	No	Yes (2x)	No	No	No	Very low
Taurococholic acid (TCA)	No	Yes (2x)	No	No	No	Very low
Taurodeoxycholic acid (TDCA)	No	Yes (2x)	No	No	No	Very low
Taurochenodeoxycholic acid (TCDCA)	No	Yes (2x)	No	No	No	Very low
Tauroursodeoxycholic acid (TUDCA)	No	Yes (2x)	No	No	No	Very low
Taurolithocholic acid (TLCA)	No	Yes (2x)	No	No	No	Very low

Abbreviations: GRADE, the Grading of Recommendations Assessment, Development and Evaluation. Downgrading of up to three levels. “No” indicates that an outcome was not downgraded. If “Yes,” then the levels of downgrading are indicated in the brackets.

Table S5. Subgroup analysis of circulating bile acids compared with plasma bile acids in MASLD patients.

**Characteristics**	**Serum bile acids**				**Plasma bile acids**			
	**Study**	**SMD (95% CI)**	***P*** †	***I***^{2}	**Study**	**SMD (95% CI)**	***P*** †	***I***^{2}
Total bile acids	11	**1.02 (0.60, 1.43)**	**<0.001**	96%	1	1.11 (0.75, 1.47)	-	-
Total unconjugated bile acids	6	**0.90 (0.37, 1.43)**	**<0.001**	91%	1	0.69 (0.34, 1.04)	-	-
Total conjugated bile acids	6	**0.81(0.47, 1.16)**	**<0.001**	79%	1	0.83 (0.47, 1.18)	-	-
Total primary bile acids	6	**1.13 (0.49, 1.77)**	**<0.001**	95%	1	0.71 (0.23, 1.20)	-	-
Total secondary bile acids	6	0.48 (-0.33, 1.29)	0.25	97%	1	-0.43 (-0.91, 0.04)	-	-
Cholic acid (CA)	13	**0.51 (0.18, 0.83)**	**0.002**	90%	2	0.27 (-0.01, 0.54)	0.06	0%
Deoxycholic acid (DCA)	13	0.26 (-0.08, 0.61)	0.14	91%	2	**0.42 (0.14, 0.69)**	**0.003**	0%
Chenodeoxycholic acid (CDCA)	13	**0.88 (0.51, 1.25)**	**<0.001**	92%	2	-0.08 (-1.19, 1.04)	0.89	93%
Ursodeoxycholic acid (UDCA)	11	**0.96 (0.51, 1.40)**	**<0.001**	94%	1	-0.11 (-0.44, 0.23)	-	-
Lithocholic acid (LCA)	9	0.24 (-0.34, 0.81)	0.42	95%	1	-0.46 (-0.80, -0.11)	-	-
Glycocholic acid (GCA)	11	**0.62 (0.33, 0.91)**	**<0.001**	85%	2	**0.83 (0.55, 1.12)**	**<0.001**	0%
Glycodeoxycholic acid (GDCA)	11	0.03 (-0.44, 0.51)	0.90	95%	2	**0.40 (0.01, 0.79)**	**0.04**	45%
Glycochenodeoxycholic acid (GCDCA)	11	**0.61 (0.30, 0.92)**	**<0.001**	87%	2	**0.76 (0.47, 1.04)**	**<0.001**	0%
Glycoursodeoxycholic acid (GUDCA)	9	**0.61 (0.27, 0.95)**	**<0.001**	86%	1	0.86 (0.51, 1.21)	-	-
Glycolithocholic acid (GLCA)	8	-0.13 (-0.83, 0.56)	0.71	96%	1	0.25 (-0.02, 0.53)	0.07	0%
Taurocholic acid (TCA)	10	**0.80 (0.47, 1.13)**	**<0.001**	88%	2	**0.73 (0.45, 1.01)**	**<0.001**	0%
Taurodeoxycholic acid (TDCA)	10	0.12 (-0.33, 0.57)	0.60	94%	2	**0.42 (0.15, 0.70)**	**0.003**	1%
Taurochenodeoxycholic acid (TCDCA)	10	**0.63 (0.30, 0.96)**	**<0.001**	88%	2	**0.79 (0.50, 1.07)**	**<0.001**	0%
Tauroursodeoxycholic acid (TUDCA)	9	**0.53 (0.11, 0.96)**	**0.01**	90%	1	0.54 (0.20, 0.89)	-	-
Taurolithocholic acid (TLCA)	7	0.28 (-0.33, 0.88)	0.37	91%	2	0.08 (-0.20, 0.35)	0.59	0%

Abbreviations: MASLD, metabolic dysfunction-associated steatotic liver disease; SMD, standardized mean difference; CI, confidence interval. † *P* denotes the *p*-value for statistical significance based on the Z test.

Table S6. Subgroup analysis of circulating bile acid levels in different diagnostic methods for MASLD patients.

**Characteristics**	Histology				**Others**			
	**Study**	**SMD (95% CI)**	***P*†**	***I*²**	**Study**	**SMD (95% CI)**	***P*†**	***I*²**
Total bile acids	8	**1.25 (0.54, 1.95)**	**<0.001**	95%	4	**0.85 (0.31, 1.38)**	**0.002**	95%
Total unconjugated bile acids	5	**0.63 (0.37, 0.89)**	**<0.001**	63%	2	1.64 (-0.77, 4.06)	0.18	98%
Total conjugated bile acids	5	**0.84(0.46, 1.21)**	**<0.001**	81%	2	**0.76 (0.17, 1.35)**	**0.01**	73%
Total primary bile acids	5	**0.73 (0.37, 1.09)**	**<0.001**	69%	2	1.67 (-1.37, 4.70)	0.28	99%
Total secondary bile acids	5	**0.83 (0.05, 1.61)**	**0.04**	93%	2	-0.93 (-3.21, 1.35)	0.42	98%
Cholic acid (CA)	9	**0.41 (0.09, 0.74)**	**0.01**	83%	6	**0.55 (-0.01, 1.10)**	**0.05**	93%
Deoxycholic acid (DCA)	9	**0.49 (0.16, 0.81)**	**0.004**	83%	6	-0.08 (-0.70, 0.53)	0.79	94%
Chenodeoxycholic acid (CDCA)	9	**0.53 (0.16, 0.89)**	**0.005**	86%	6	**1.03 (0.29, 1.78)**	**0.007**	96%
Ursodeoxycholic acid (UDCA)	6	**0.46 (0.04, 0.88)**	**0.03**	87%	6	**1.18 (0.39, 1.98)**	**0.003**	96%
Lithocholic acid (LCA)	5	0.47 (-0.14, 1.09)	0.13	92%	5	-0.20 (-1.29, 0.89)	0.72	97%
Glycocholic acid (GCA)	7	**0.71 (0.41, 1.02)**	**<0.001**	75%	6	**0.55 (0.10, 1.01)**	**0.02**	89%
Glycodeoxycholic acid (GDCA)	7	**0.44 (0.09, 0.79)**	**0.01**	82%	6	-0.51 (-1.31, 0.29)	0.21	96%
Glycochenodeoxycholic acid (GCDCA)	7	**0.68 (0.32, 1.03)**	**<0.001**	82%	6	**0.56 (0.13, 0.99)**	**0.01**	88%
Glycoursodeoxycholic acid (GUDCA)	4	**0.89 (0.52, 1.26)**	**<0.001**	64%	6	**0.44 (0.06, 0.82)**	**0.02**	84%
Glycolithocholic acid (GLCA)	5	**0.51 (0.03, 0.99)**	**0.04**	83%	5	-0.76 (-1.82, 0.30)	0.07	97%
Taurocholic acid (TCA)	6	**0.65 (0.34, 0.95**	**<0.001**	71%	6	**0.88 (0.37, 1.38)**	**<0.001**	91%
Taurodeoxycholic acid (TDCA)	6	**0.47 (0.17, 0.78)**	**0.003**	72%	6	-0.26 (-0.97, 0.46)	0.49	96%
Taurochenodeoxycholic acid (TCDCA)	6	**0.72 (0.45, 0.99)**	**<0.001**	62%	6	**0.53 (0.03, 1.03)**	**0.04**	91%
Tauroursodeoxycholic acid (TUDCA)	5	**0.55 (0.13, 0.97)**	**0.01**	83%	5	0.44 (-0.29, 1.17)	0.24	93%
Taurolithocholic acid (TLCA)	5	0.30 (-0.07, 0.66)	0.11	69%	4	0.02 (-0.88, 0.92)	0.96	93%

Abbreviations: MASLD, metabolic dysfunction-associated steatotic liver disease; SMD, standardized mean difference; CI, confidence interval. † *P* denotes the *p*-value for statistical significance based on the Z test.
